# Relação entre os Sinais de Strain do Ventrículo Direito no Eletrocardiograma e Níveis de Biomarcadores Associados à Gravidade da Pneumonia por COVID-19

**DOI:** 10.36660/abc.20200724

**Published:** 2021-06-25

**Authors:** Veli Polat, Evin Bozcali, Kadriye Kart Yasar, Hayat Kumbasar Karaosmanoglu, Ibrahim Faruk Akturk

**Affiliations:** 1 Departamento de Cardiologia Bakirkoy Dr. Sadi Konuk Training and Research Hospital Istambul Turquia Departamento de Cardiologia, Bakirkoy Dr. Sadi Konuk Training and Research Hospital, Istambul - Turquia; 2 Departamento de Cardiologia Prof. Dr. Cemil Tascioglu City Hospital Istambul Turquia Departamento de Cardiologia, Prof. Dr. Cemil Tascioglu City Hospital, Istambul - Turquia; 3 Departamento de Doenças Infecciosas e Microbiologia Clínica Bakirkoy Dr. Sadi Konuk Training and Research Hospital Istambul Turquia Departamento de Doenças Infecciosas e Microbiologia Clínica, Bakirkoy Dr. Sadi Konuk Training and Research Hospital, Istambul - Turquia

**Keywords:** COVID-19, Betacoronavirus, Doenças Cardiovasculares, Função Ventricular Direita, Estresse, Eletrocardiografia/métodos, Biomarcadores, Pneumonia/complicações

## Abstract

**Fundamento:**

A nova doença por coronavírus (COVID-19) pode levar a uma enfermidade grave e causar a morte. Sabe-se que a COVID-19 afeta o sistema cardiovascular. A detecção precoce da progressão para um estágio grave da doença que afeta o sistema cardiovascular pode desempenhar um papel crítico no tratamento da COVID-19.

**Objetivos:**

Explorar a possível relação entre a pneumonia por COVID-19 e os achados de *strain* do ventrículo direito no eletrocardiograma (ECG).

**Métodos:**

Foi realizado um estudo retrospectivo de 141 pacientes hospitalizados com COVID-19. A correlação de Spearman e as análises de regressão logística foram aplicadas para avaliar as relações entre as manifestações de *strain* ventricular direito na ECG e os níveis de biomarcadores e outros achados laboratoriais e de imagem do tórax. O nível de significância foi considerado estabelecido como *p* < 0,05.

**Resultados:**

Os sinais de ECG de estresse ventricular direito foram significativamente mais frequentes e os níveis de fibrinogênio, PCR e ferritina foram significativamente mais elevados em pacientes com COVID-19 com níveis elevados de hs-cTnI, procalcitonina e dímero-D. A análise univariada mostrou que existem relações significativas entre a presença de pneumonia bilateral, a maioria dos sinais eletrocardiográficos de *strain* ventricular direito e lesão cardíaca e biomarcadores inflamatórios e trombóticos. A análise multivariada revelou que o supradesnivelamento do segmento ST em V1 e padrão S_1_Q_3_T_3_ são preditores independentes de lesão cardíaca ( *odds ratio* =0,23; IC95%, 0,06 a 0,90; p=0,035) e níveis elevados de procalcitonina ( *odds ratio* =0,19; IC 95%, 0,06 a 0,62; p=0,006), respectivamente.

**Conclusão:**

Os achados do presente estudo sugerem que a dano cardíaco direito é prevalente na COVID-19. Além disso, nosso estudo demonstra o valor clínico do ECG na avaliação e monitoramento de pacientes com pneumonia por COVID-19.

## Introdução

A nova pneumonia por coronavírus (COVID-19) de 2019, surgida pela primeira vez em Wuhan, na província chinesa de Hubei, em dezembro de 2019, se espalhou rapidamente pelo mundo. Embora os sintomas respiratórios sejam sinais clínicos primários de COVID-19, vários pacientes também desenvolvem lesão cardiovascular, que é predominantemente detectada como um aumento nos testes de troponina cardíaca de alta sensibilidade.^1^ Além disso, foi demonstrado que a pneumonia por COVID-19 tem uma pior evolução clínica em pacientes com doença cardiovascular.^2^ Nesse contexto, revelar as maneiras pelas quais o novo coronavírus afeta o sistema cardiovascular é crucial para o tratamento desses pacientes de maneira oportuna e eficiente e para a redução de desfechos nefastos, como a morte.

No presente estudo, examinamos as manifestações anormais do eletrocardiograma (ECG) na COVID-19. Especificamente, nosso objetivo foi investigar a possível relação entre a gravidade da pneumonia por COVID-19 e os achados de *strain* do ventrículo direito no ECG. Além disso, objetivamos explorar possíveis relações entre as anormalidades eletrocardiográficas, dano cardíaco, bem como biomarcadores elevados de inflamação e trombose em pacientes com COVID-19.

## Materiais e métodos

### Pacientes do estudo

Este estudo retrospectivo, de centro único e observacional, foi conduzido no Bakirkoy Dr. Sadi Konuk Training and Research Hospital. Entre 1º de abril e 30 de abril de 2020, 141 pacientes consecutivos hospitalizados com diagnóstico de pneumonia por COVID-19, de acordo com as orientações provisórias da Organização Mundial da Saúde,^3^ foram incluídos no presente estudo. O diagnóstico de infecção por COVID-19 também foi confirmado pelo resultado positivo da análise por reação em cadeia da polimerase da transcrição reversa em tempo real de amostras de esfregaço nasal ou faríngeo do paciente. Os ECGs dos pacientes foram obtidos na admissão hospitalar. Todos os pacientes do estudo eram adultos (maiores de 18 anos) e o diagnóstico de embolia pulmonar foi excluído pelos achados de tomografia computadorizada (TC) de tórax com contraste em todos os pacientes.

### Coleta de dados

Os dados demográficos, epidemiológicos, clínicos e radiológicos, bem como as comorbidades e os achados laboratoriais na admissão, foram registrados nos prontuários eletrônicos de cada paciente do estudo. Os dados laboratoriais incluíram a troponina I cardíaca de alta sensibilidade (hs-cTnI), creatina quinase MB (CK-MB), hemograma completo, proteína-C reativa (PCR), procalcitonina, fibrinogênio, dímero-D, ferritina, testes de função renal e hepática. Todos os pacientes foram acompanhados clinicamente até 25 de maio de 2020. Dois investigadores controlaram todos os dados do estudo.

Níveis elevados de hs-cTnI, que é o sinal de dano cardíaco, foram considerados quando acima do percentil 99 do limite superior de referência. Os níveis de procalcitonina e dímero-D foram considerados elevados quando ≥ 0,5 ng/mL e ≥ 0,5 µg/mL, respectivamente.^4^

### Avaliação Eletrocardiográfica

Os ECGs de 12 derivações obtidos no momento da admissão foram interpretados independentemente em termos de sinais de *strain* do ventrículo direito por dois cardiologistas experientes que desconheciam os dados dos pacientes. Os sinais de ECG foram determinados com base nas manifestações do ECG devido ao estresse ventricular direito secundário à hipertensão pulmonar no embolismo pulmonar.^5 , 6^ Os seguintes sinais do ECG de *strain* ventricular direito foram avaliados: (1) taquicardia sinusal (frequência cardíaca >100 batimentos/minuto),^5^ (2) bloqueio de ramo direito (BRD)^5^ incompleto ou completo, (3) inversão da onda T nas derivações V1 a V4,^5^ (4) padrão S_1_Q_3_T_3,_^5^ (5) morfologia Qr na derivação V1 (existência de Onda Q proeminente ≥ 0,2 milivolts quando a duração do QRS é <120 milissegundos)^6^ , (6) elevação do segmento ST ≥ 0,1 milivolts na derivação V1.^6^

### Análise Estatística

A estatística descritiva foi realizada utilizando-se média ± desvio padrão (DP) para distribuição normal ou mediana e intervalos interquartis (IIQ) para variáveis contínuas distribuídas assimetricamente e porcentagem para variáveis categóricas. A distribuição das variáveis foi avaliada pelo teste de Kolmogorov-Smirnov. As variáveis contínuas foram comparadas pelo teste *t* de amostras independentes quando distribuídas normalmente ou pelo teste U de Mann-Whitney quando não distribuídas normalmente. As variáveis categóricas foram comparadas com o teste do qui-quadrado (X^2^) ou o teste exato de Fisher, quando apropriado. As correlações entre os níveis de hs-cTnI, procalcitonina, dímero-D, PCR, fibrinogênio, ferritina e contagem de neutrófilos foram avaliadas pela análise do coeficiente de correlação de Spearman. A análise de regressão logística univariada foi realizada para testar a relação entre as variáveis e os níveis elevados de hs-cTnI, procalcitonina e dímero-D. Além disso, os valores dos *odds ratios* e intervalos de confiança (IC) de 95% foram calculados para cada variável. A análise de regressão logística multivariada foi realizada para avaliar os preditores independentes de dano cardíaco (níveis elevados de hs-cTnI), níveis elevados de procalcitonina e de dímero-D. As variáveis que foram significativas na análise univariada foram incluídas na análise multivariada. Um valor de p<0,05 foi considerado estatisticamente significativo. Todas as análises estatísticas foram realizadas com o software IBM SPSS, versão 26.0.

## Resultados

Foram analisados no presente estudo cento e quarenta e um pacientes consecutivos hospitalizados com pneumonia por COVID-19. A mediana da idade dos pacientes do estudo foi de 64 anos (variação, 26-91 anos) e 84 (59,6%) eram do sexo masculino. Cinco pacientes morreram durante a hospitalização e 136 pacientes receberam alta. Comorbidades, sinais eletrocardiográficos de *strain* ventricular direito, achados radiográficos e a evolução clínica de todos os pacientes do estudo são mostrados na Tabela 1 .

**Tabela 1:** – Comorbidades, evolução clínica, achados eletrocardiográficos e radiográficos de pacientes com pneumonia por COVID-19

	Pacientes (n=141)
Tabagismo	21 (14,9%)
Comorbidades	
Hipertensão	44 (31,2%)
Diabetes	36 (25,5%)
Doença arterial coronariana	27 (19,1%)
Doença pulmonar obstrutiva crônica	5 (3,5%)
Malignidade	2 (1,4%)
Sinais do ECG	
Taquicardia sinusal	87 (61,7%)
BRD incompleto	62 (44,0%)
BRD Completo	28 (19,9%)
Inversão da onda T in V1 a V4	37 (26,2%)
Padrão S_1_Q_3_T_3_	52 (36,9%)
Qr em V_1_	35 (24,8%)
Supradesnivelamento de ST em V_1_	54 (38,3%)
Achados da tomografia computadorizada do tórax	7 (5%)
Pneumonia unilateral	55 (39,0%)
Pneumonia bilateral	86 (61,0%)
Desfecho clínico	35 (24,8%)
Morte hospitalar	5 (3,5%)

*ECG: eletrocardiograma; BRD: Bloqueio do ramo direito.*

### Características clínicas e eletrocardiográficas

A Tabela 2 mostra um resumo da comparação de dados demográficos, sinais de ECG, achados da TC, desfecho clínico e dados laboratoriais, ao categorizar em termos de níveis elevados ou normais de hs-cTnI, procalcitonina e dímero-D em pacientes com pneumonia por COVID-19. Tabagismo, gênero e presença de comorbidades foram semelhantes entre os pacientes com níveis elevados ou normais de hs-cTnI, procalcitonina e dímero-D.

**Tabela 2:** – Comparação das características dos pacientes com pneumonia por COVID-19 de acordo com níveis elevados ou normais de hs-cTnI, procalcitonina e dímero-D

Características	hs-cTnI normal (n = 69)	hs-cTnI elevada (n = 72)	p Valor	Procalcitonina < 0.5 ng/mL (n = 74)	Procalcitonina ≥ 0.5 ng/mL (n = 67)	p Valor	Dímero-D < 0.5 µg/mL (n = 39)	Dímero-D ≥ 0.5 µg/mL (n = 102)	p Valor
Idade	62,6 ± 12,3	63,4 ± 12,5	0,69^†^	60,1 ± 12,1	66,3 ± 11,1	0,003^†^	57,0 ± 12,6	65,3 ± 11,5	< 0,001^†^
Sexo feminino	29 (42%)	28 (38,9%)	0,70^∗^	29 (39,2%)	28 (41,8%)	0,75^∗^	13 (33,3%)	44 (43,1%)	0,289^∗^
Sinais do ECG									
Taquicardia sinusal	34 (49,3%)	53 (73,6%)	0,003^∗^	30 (40,5%)	57 (85,1%)	< 0,001^∗^	9 (23,1%)	78 (76,5%)	< 0,001^∗^
BRD incompleto	21 (30,4%)	41 (56,9%)	0,002^∗^	28 (37,8%)	34 (50,7%)	0,123^∗^	16 (41%)	46 (45,1%)	0,66^∗^
BRD completo	8 (11,6%)	20 (27,8%)	0,016^∗^	4 (5,4%)	24 (35,8%)	< 0,001^∗^	2 (5,1%)	26 (25,5%)	0,007^∗^
Inversão de T em V1 a V4	3(4,3%)	34 (47,2%)	< 0,001^∗^	4 (5,4%)	33(49,3%)	< 0,001^∗^	1 (2,6%)	36 (35,3%)	< 0,001^∗^
Padrão S_1_Q_3_T_3_	14 (20,3%)	38 (52,8%)	< 0,001^∗^	8 (10,8%)	44 (65,7%)	< 0,001^∗^	2 (5,1%)	50 (49%)	< 0,001^∗^
Qr em V_1_	1 (1,4%)	34 (47,2%)	< 0,001^∗^	3 (4,1%)	32 (47,8%)	< 0,001^∗^	1 (2,6%)	34 (33,3%)	< 0,001^∗^
Supradesnivelamento de ST em V_1_	5 (7,2%)	49 (68,1%)	< 0,001^∗^	5 (6,8%)	49 (73,1%)	< 0,001^∗^	0 (0,0%)	54 (52,9%)	< 0,001^∗^
Achados de TC de tórax									
Pneumonia unilateral	40 (58%)	15 (20,8%)	< 0,001^∗^	52 (70,3%)	3 (4,5%)	< 0,001^∗^	31 (79,5%)	24 (23,5%)	< 0,001^∗^
Pneumonia bilateral	29 (42%)	57 (79,2%)		22 (29,7%)	64 (95,5%)		8 (20,5%)	78 (76,5%)	
Desfecho clínico									
Morte hospitalar	0 (0,0%)	5 (6,9%)	0,026^γ^	0 (0,0%)	5 (7,5%)	0,017^γ^	0 (0,0%)	5 (4,9%)	0,322^γ^
Dados laboratoriais									
Contagem de leucócitos ×10^3^/μL	6,6 (5,6-7,8)	6,9 (5,3-8,9)	0,66^‡^	6,5 (5,3-8,2)	7,3 (5,7-8,6)	0,104^‡^	6,8 (5,4-7,7)	6,7 (5,6-6,6)	0,269^‡^
Contagem de neutrófilos ×10^3^/μL	4,9 (3,3-5,9)	4,7 (3,9 – 6,8)	0,22^‡^	4,7 (3,2-5,7)	5,4 (4-7,8)	0,004^‡^	4,9 (3,2-5,8)	4,9 (3,8-6,8)	0,203^‡^
Relação Neutrófilos/ Leucócitos, %	70,5 ± 8,9	74,0 ± 10,2	0,033^†^	69,6 ± 9,2	75,3 ± 9,5	< 0,001^†^	69,4 ± 9,4	73,4± 9,7	0,03^†^
Contagem de linfócitos ×10^3^/μL	1,1 (0,9- 1,3)	0,9 (0,8-1,2)	0,006^‡^	1,05 (0,9-1,1)	0,96 (0,9-1,2)	0,019^‡^	1,10 (0,9-1,6)	0,97 (1-1,2)	0,009^‡^
Relação Linfócitos/ Leucócitos, %	15,6 (13,6 -20)	15 (10-19,7)	0,06^‡^	16,7 (14,3-20)	13,6 (9,5 -19)	0,003^‡^	17 (14-17)	15 (10-18)	0,007^‡^
Plaquetas ×10^3^/μL	220 (168 – 256)	221 (163-285)	0,98^‡^	219 (167-256)	220 (166-306)	0,82^‡^	218 (163-243)	220 (167-291)	0,411^‡^
Creatinina (mg/dL)	0,86 (0,7-1)	0,98 (0,8 -1,2)	0,16^‡^	0,86 (0,7-1,1)	0,98 (0,8-1,3)	0,09^‡^	0,83 (0,7-1)	0,97 (0,8-1,2)	0,13^‡^
ALT (U/L)	31 (25-45)	31,5 (20-49)	0,74^‡^	30 (21-45)	32 (23-47)	0,55^‡^	31 (25-43)	31 (21-45)	0,32^‡^
AST (U/L)	30 (24-38)	35,5 (26-47)	0,11^‡^	30 (23-39)	36 (26-46)	0,23^‡^	30 (23-40)	34 (26-44)	0,37^‡^
Fibrinogênio, mg/dL	472 (413-542)	590 (505-736)	< 0,001^‡^	449 (409-507)	626 (550-740)	< 0,001^‡^	415 (396-428)	577 (505-683)	< 0,001^‡^
Dímero-D, µg/mL	0,45 (0,2-0,9)	1,40 (0,7-3,7)	< 0,001^‡^	0,41 (0,2-0,8)	2,04 (1-3,8)	< 0,001^‡^	0,24 (0,2-0,3)	1,20 (0,7-2,8)	< 0,001^‡^
PCR, mg/L	58 (44-88)	136 (89-203)	< 0,001^‡^	54 (43-87)	153 (153-204)	< 0,001^‡^	49 (41-61)	114 (77-179)	< 0,001^‡^
Ferritina, µg/L	294 (236-400)	612 (397-975)	< 0,001^‡^	282 (232- 382)	662 (450-980)	< 0,001^‡^	254 (226-292)	532 (356-806)	< 0,001^‡^
Procalcitonina, ng/mL	0,06 (0,02-0,38)	2,76 (0,31-6,69)	< 0,001^‡^	0,055 (0,02-0,07)	2,76 (1-7,7)	< 0,001^‡^	0,05 (0,02-0,06)	0,96 (0,07-5,30)	< 0,001^‡^
CK-MB, ng/mL	3 (2-3)	8,5 (5-15,5)	< 0,001^‡^	3 (2-4)	9 (4-16)	< 0,001^‡^	3 (2-4)	5,5 (3-12)	< 0,001^‡^
hs-cTnI, ng/L	15 (12-16)	62,5 (26-348)	< 0,001^‡^	16 (13-18)	67 (18-356)	< 0,001^‡^	16 (13-16)	31 (16-173)	< 0,001^‡^

*Os dados são apresentados como média ± DP ou mediana (IIQ) ou No. (%). ECG: eletrocardiograma; hs-cTnI: troponina I cardíaca de alta sensibilidade; TC: tomografia computadorizada; ALT: alanina aminotransferase; AST: aspartato aminotransferase; PCR: proteína C-reativa; CK-MB: creatinina quinase-MB. †Comparado utilizando o teste t de amostra independente, ‡Comparado utilizando o teste U de Mann-Whitney, ∗Comparado utilizando o teste X2, γComparado utilizando o teste exato de Fisher.*

Nos pacientes com níveis elevados de hs-cTnI, procalcitonina e dímero-D, os sinais de ECG de estresse ventricular direito, incluindo taquicardia sinusal, BRD total, inversão da onda T em V1-V4, padrão S_1_Q_3_T_3_, morfologia Qr na derivação V1 e supradesnivelamento do segmento ST em V1 foi significativamente mais prevalente em comparação com os pacientes com níveis normais de hs-cTnI, procalcitonina e dímero-D. A proporção de pneumonia bilateral com base nos achados da TC de tórax foi significativamente maior em pacientes com níveis elevados de hs-cTnI, procalcitonina e dímero-D. Pacientes com níveis elevados de hs-cTnI, procalcitonina e dímero-D também apresentaram níveis significativamente mais elevados de fibrinogênio, PCR, ferritina, CK-MB e a razão neutrófilos-leucócitos mais alta, enquanto mostravam contagem de linfócitos significativamente mais baixa ( Tabela 2 ).

A mortalidade hospitalar foi significativamente maior nos pacientes com níveis elevados de hs-cTnI e procalcitonina. O BRD incompleto foi significativamente frequente nos pacientes que apresentavam apenas dano cardíaco. Pacientes com níveis elevados de procalcitonina apresentaram contagem de neutrófilos significativamente mais alta do que aqueles com níveis normais de procalcitonina. Os pacientes com níveis elevados de procalcitonina e dímero-D eram significativamente mais velhos e a relação linfócitos/leucócitos era significativamente menor ( Tabela 2 ).

Além disso, os pacientes com dano cardíaco apresentaram níveis significativamente mais elevados de dímero-D e procalcitonina do que aqueles sem danos. Os níveis de dímero-D e hs-cTnI estavam significativamente aumentados em pacientes com níveis elevados de procalcitonina em comparação com pacientes com níveis normais de procalcitonina. Os níveis de procalcitonina e hs-cTnI também estavam significativamente mais elevados em pacientes com níveis elevados de dímero-D em comparação com aqueles com níveis normais de dímero-D ( Tabela 2 ).

### Análises de regressão logística univariada e multivariada

A análise de regressão logística univariada mostrou que taquicardia sinusal, BRD incompleto, BRD completo, inversão da onda T em V1-V4, padrão S_1_Q_3_T_3_, morfologia Qr em V1, supradesnivelamento do segmento ST em V1, presença de pneumonia bilateral, contagem de neutrófilos, PCR, procalcitonina, ferritina, fibrinogênio, dímero-D e CK-MB foram significativamente associados ao dano cardíaco em pacientes com pneumonia por COVID-19 ( Tabela 3 ). Entretanto, na análise de regressão logística multivariada, apenas o supradesnivelamento do segmento ST em V1 foi determinado como um preditor independente de dano cardíaco em pacientes com pneumonia por COVID-19 ( *odds ratio* = 0,23; IC 95%, 0,06 a 0,90; p=0,035).

**Tabela 3:** – Determinantes do dano cardíaca em pacientes com COVID-19 por análises de regressão logística univariada e multivariada

	Univariada		Multivariada	

	OR (95% CI)	p Value	*OR* (95% CI)	p Valor
Taquicardia sinusal	0,35 (0,17-0,70)	0,003		
BRD incompleto	0,33 (0,17-0,66)	0,002		
BRD completo	0,34 (0,14-0,84)	0,019		
Inversão T em V1-V4	0,05 (0,01-0,18)	< 0,001		
Padrão S1Q3T3	0,23 (0,11-0,48)	< 0,001		
Qr em V1	0,02 (0,00-0,12)	< 0,001		
Supradesnivelamento do ST em V1	0,04 (0,01-0,10)	< 0,001	0,23 (0,06-0,90)	0,035
Presença de pneumonia bilateral	0,19 (0,09-0,40)	< 0,001		
Contagem de neutrófilos	1,04 (1,00-1,08)	0,036		
Fibrinogênio, mg/dL	1,01 (1,01-1,01)	< 0,001		
Dímero-D, µg/mL	3,14 (1,91-5,17)	< 0,001		
PCR, mg/L	1,02 (1,01-1,03)	< 0,001		
Ferritina, µg/L	1,01 (1,00-1,01)	< 0,001		
Procalcitonina, ng/mL	2,03 (1,45-2,83)	< 0,001		
CK-MB, ng/mL	2,09 (1,58-2,76)	< 0,001		

*OR: Odds ratio; IC: intervalo de confiança; BRD: bloqueio de ramo direito; PCR: proteína C-reativa.*

A idade dos pacientes, BRD completo, inversão da onda T em V1-V4, padrão S_1_Q_3_T_3_, morfologia Qr em V1, supradesnivelamento do segmento ST em V1, presença de pneumonia bilateral, relação linfócitos/leucócitos, contagem de neutrófilos, relação neutrófilos/leucócitos, níveis de PCR, ferritina, fibrinogênio, dímero-D, hs-cTnI, e CK-MB foram significativamente associados com os níveis elevados de procalcitonina na análise de regressão logística univariada. A análise de regressão logística multivariada revelou que o padrão S_1_Q_3_T_3_ ( *odds ratio* = 0,19; IC 95%, 0,06 a 0,62; p = 0,006), contagem de neutrófilos ( *odds ratio* = 1,35; IC 95%, 1,07 a 1,71; p = 0,011) e a presença de pneumonia bilateral ( *odds ratio* = 0,15; IC 95%, 0,03 a 0,84; p = 0,031) foram independentemente associadas aos níveis elevados de procalcitonina ( Tabela 4 ).

**Tabela 4:** – Determinantes dos níveis elevados de procalcitonina (≥ 0,5 ng/mL) em pacientes com COVID-19 por análises de regressão logística univariada e multivariada

	Univariada		Multivariada	

	OR (95% CI)	p Valor	OR (95% CI)	p Valor
Idade	1,04 (1,01-1,08)	0,004		
BRD completo	0,10 (0,03-0,32)	< 0,001		
Inversão de T em V_1_-V_4_	0,06 (0,02-0,18)	< 0,001		
Padrão S_1_Q_3_T_3_	0,06 (0,03-0,15)	< 0,001	0,19 (0,06-0,62)	0,006
Qr em V_1_	0,05 (0,01-0,16)	< 0,001		
Supradesnivelamento de ST em V_1_	0,03 (0,01-0,08)	< 0,001		
Presença de pneumonia bilateral	0,02 (0,01-0,07)	< 0,001	0,15 (0,03-0,84)	0,031
Contagem de neutrófilos	1,27 (1,09-1,48)	0,002	1,35 (1,07-1,71)	0,011
Relação Neutrófilos/Leucócitos	1,07 (1,03-1,11)	0,001		
Relação Linfócitos/Leucócitos	0,93 (0,88-0,98)	0,009		
Fibrinogênio, mg/dL	1,02 (1,01-1,02)	< 0,001		
Dímero-D, µg/mL	8,64 (3,67-20,35)	< 0,001		
PCR, mg/L	1,03 (1,02-1,04)	< 0,001		
Ferritina, µg/L	1,01 (1,01-1,01)	< 0,001		
CK-MB, ng/mL	1,35 (1,18-1,53)	< 0,001		
hs-cTnI, ng/L	1,02 (1,01-1,02)	< 0,001		

*OR: Odds ratio; IC: intervalo de confiança; BRD: bloqueio de ramo direito; PCR: proteína C-reativa.*

Pela análise univariada, a idade dos pacientes, a presença de pneumonia bilateral, a relação linfócito-leucócito, contagens de neutrófilos e linfócitos, níveis de PCR, ferritina, fibrinogênio, procalcitonina, hs-cTnI, CK-MB e presença dos sinais do ECG, incluindo BRD completo, inversão de onda T em V1-V4, padrão S_1_Q_3_T_3_, e morfologia Qr em V1 foram significativamente associados com os níveis elevados de dímero-D ( Tabela 5 ). No entanto, entre esses determinantes, apenas o fibrinogênio mostrou ser um preditor independente dos níveis elevados de dímero-D na análise multivariada ( *odds ratio* = 1,05; IC 95%, 1,03 a 1,07; p <0,001).

**Tabela 5:** – Determinantes dos níveis elevados de dímero-D (≥ 0,5 µg / mL) em pacientes com COVID-19 por análises de regressão logística univariada e multivariada

	Univariada		Multivariada	

	OR (95% CI)	p Valor	OR (95% CI)	p Valor
Idade	1,06 (1,02-1,10)	0,001		
BRD completo	0,16 (0,04-0,70)	0,015		
Inversão de T em V1-V4	0,05 (0,01-0,37)	0,003		
Padrão S_1_Q_3_T_3_	0,06 (0,01-0,25)	< 0,001		
Qr em V1	0,05 (0,01-0,40)	0,004		
Presença de Pneumonia bilateral	0,08 (0,03-0,20)	< 0,001		
Contagem de neutrófilos	1,04 (1,00-1,09)	0,033		
Contagem de linfócitos	0,41 (0,17-0,96)	0,041		
Relação Linfócitos/ Leucócitos	0,93 (0,88-0,98)	0,007		
Fibrinogênio, mg/dL	1,04 (1,02-1,06)	< 0,001	1,05 (1,03-1,07)	< 0,001
Procalcitonina, ng/mL	17,73 (2,82-111,30)	0,002		
PCR, mg/L	1,05 (1,03-1,07)	< 0,001		
Ferritina, µg/ L	1,01 (1,01-1,02)	< 0,001		
CK-MB, ng/mL	1,34 (1,12-1,61)	0,001		
hs-cTnI, ng/L	1,10 (1,03-1,18)	0,006		

*OR: Odds ratio; IC: intervalo de confiança; BRD: bloqueio de ramo direito; PCR: proteína C-reativa.*

A análise de correlação de Spearman também revelou que a contagem de neutrófilos, níveis de hs-cTnI, procalcitonina, dímero-D, PCR, fibrinogênio e ferritina estavam significativa e positivamente correlacionados entre si ( Tabela 6 ).

**Tabela 6:** – Correlações de Spearman dos biomarcadores entre si em pacientes com COVID-19

	hs-cTnI		Procalcitonina		Dímero-D		PCR		Contagem de neutrófilos		Fibrinogênio		Ferritina	

	r	p	r	p	r	p	r	p	r	p	r	p	r	p
hs-cTnI	1	,	0,666	< 0,001	0,633	< 0,001	0,599	< 0,001	0,175	0,04	0,618	< 0,001	0,625	< 0,001
Procalcitonina	0,666	< 0,001	1	,	0,776	< 0,001	0,811	< 0,001	0,213	0,01	0,748	< 0,001	0,841	< 0,001
Dímero-D	0,633	< 0,001	0,776	< 0,001	1	,	0,821	< 0,001	0,172	0,04	0,924	< 0,001	0,842	< 0,001
PCR	0,599	< 0,001	0,811	< 0,001	0,821	< 0,001	1	,	0,271	0,001	0,811	< 0,001	0,962	< 0,001
Contagem de neutrófilos	0,175	0,04	0,213	0,01	0,172	0,04	0,271	0,001	1	,	0,174	0,04	0,222	0,008
Fibrinogênio	0,618	< 0,001	0,748	< 0,001	0,924	< 0,001	0,811	< 0,001	0,174	0,04	1	,	0,832	< 0,001
Ferritina	0,625	< 0,001	0,841	< 0,001	0,842	< 0,001	0,962	< 0,001	0,222	0,008	0,832	< 0,001	1	,

*PCR: proteína C-reativa; hs-cTnI: troponina I cardíaca de alta sensibilidade; r: coeficiente de correlação de Spearman.*

## Discussão

O dano cardíaco detectado pelo aumento de biomarcadores cardíacos na COVID-19 foi associado a um prognóstico fatal. Entretanto, os mecanismos fisiopatológicos subjacentes ao dano cardíaco associado à COVID-19 ainda não foram claramente demonstrados. Os possíveis mecanismos que outros estudos sugeriram para explicar o dano miocárdico causado pela COVID-19 são miocardite devido à infecção direta com SARS-CoV-2 ou secundária à resposta inflamatória sistêmica vigorosa induzida pela COVID-19, instabilidade da placa que leva à síndrome coronariana aguda e hipoxemia devido a insuficiência respiratória.^7 , 8^ Recentemente, trombose microvascular pulmonar foi relatada com dano alveolar difuso na pneumonia por COVID-19.^9 , 10^

McGonagle et al. propuseram um modelo de coagulopatia pulmonar intravascular para explicar a lesão miocárdica e a mortalidade cardiovascular na pneumonia por COVID-19. De acordo com este modelo, a COVID-19 induz a coagulopatia pulmonar intravascular difusa através de uma inflamação pulmonar difusa semelhante à síndrome de ativação macrofágica. Tanto a imunotrombose do extenso leito vascular pulmonar quanto a hipoxemia alveolar podem desencadear estresse ventricular direito ao causar hipertensão pulmonar e desempenhar um papel no desfecho fatal. Na pneumonia por COVID-19, níveis elevados de dímero-D podem refletir uma ampla microtrombose pulmonar, enquanto níveis elevados de biomarcadores cardíacos podem indicar *strain* ventricular direito desencadeado por hipertensão pulmonar.^11^ Os achados do presente estudo apoiam esse modelo. Todos os sinais de *strain* ventricular direito no ECG do estudo, exceto BRD incompleto, foram significativamente mais prevalentes em pacientes com COVID-19 com dano cardíaco e níveis elevados de procalcitonina e dímero-D. Como esperávamos, todos os sinais de *strain* ventricular direito no ECG, incluindo BRD incompleto, foram significativamente mais comuns em pacientes com COVID-19 com dano cardíaco. Também observamos associação significativa direta entre os níveis de hs-cTnI, procalcitonina e dímero-D, contagem de neutrófilos, níveis de PCR, ferritina e fibrinogênio em pacientes com pneumonia por COVID-19. Esses resultados indicam que a inflamação, trombose e dano cardíaco estão diretamente relacionados entre si em pacientes com COVID-19.

A análise de regressão univariada revelou que quatro dos sete sinais de *strain* ventricular direito no ECG estavam significativamente associados ao dano cardíaco e níveis elevados de procalcitonina e dímero-D em pacientes com pneumonia por COVID-19. Entretanto, todos os sete sinais do ECG estavam significativamente associados apenas com lesão cardíaca em pacientes com COVID-19. Esses achados sugerem que a possível causa dos níveis elevados de hs-cTnI ou lesão cardíaca em COVID-19 é o estresse ventricular direito que é induzido por hipertensão pulmonar de início súbito devido à trombose vascular pulmonar difusa desencadeada pela pneumonia por COVID-19.

A análise multivariada revelou que entre os sinais do ECG e outras variáveis, apenas o supradesnivelamento do segmento ST em V1 foi um preditor independente de lesão cardíaca em pacientes com pneumonia por COVID-19. Assim, a existência do supradesnivelamento do segmento ST em V1 no ECG à hospitalização pode indicar um mau prognóstico em pacientes com COVID-19, quando consideramos que a lesão cardíaca está associada a prognóstico ruim nesses pacientes. Além disso, a existência de supradesnivelamento do segmento ST em V1 também foi significativamente associada à procalcitonina elevada, de acordo com nossos resultados de análises de regressão. O padrão S_1_Q_3_T_3_, que é uma manifestação eletrocardiográfica bem conhecida de *strain* cardíaco direito agudo, particularmente desenvolvido devido à embolia pulmonar maciça, foi identificado como um dos preditores independentes de níveis elevados de procalcitonina em pacientes com pneumonia por COVID-19 em nossas análises de regressão multivariada. Como esperado, os outros dois preditores independentes de níveis elevados de procalcitonina em pacientes com COVID-19 foram a presença de pneumonia bilateral e contagem de neutrófilos. Como esses dois preditores estão relacionados à extensão e à gravidade da doença, sugerimos que o padrão S_1_Q_3_T_3_ também pode refletir a extensão e a gravidade da doença.

Curiosamente, não houve relação entre a idade dos pacientes e os danos cardíacos. Além disso, a análise de regressão univariada demonstrou uma relação positiva entre a idade e níveis elevados de procalcitonina e dímero-D. A análise multivariada indicou o fibrinogênio como o único preditor independente de níveis elevados de dímero-D. No entanto, nenhum dos sinais do *strain* ventricular direito no ECG foi um preditor independente de níveis elevados de dímero-D. Embora os níveis elevados de dímero-D possam ser o reflexo da trombose intravascular pulmonar difusa na COVID-19, os níveis elevados de dímero-D não são específicos e podem ocorrer devido a muitas outras condições. Portanto, podemos concluir que não encontramos uma relação independente entre os níveis elevados de dímero-D e os sinais no ECG na análise multivariada.

Muito recentemente, Ackermann et al.,^12^ publicaram um artigo que investigou os mecanismos patológicos fundamentais da infecção grave por COVID-19, examinando as alterações morfológicas e moleculares dos pulmões obtidas durante a autópsia de pacientes que morreram devido à infecção por COVID-19. Eles relataram que a COVID-19 e a influenza exibem dano alveolar difuso e infiltração de linfócitos perivasculares, embora a COVID-19 seja distinta com suas três características angiocêntricas. Essas características são a trombose vascular extensa com microangiopatia e oclusão de capilares alveolares, a angiogênese vascular via angiogênese intussusceptiva e o dano às células endoteliais possivelmente devido à invasão direta por SARS-CoV-2.^12^ Seus achados apoiam os nossos, mostrando trombose vascular pulmonar generalizada acompanhada por lesão endotelial e angiogênese causada por COVID-19. Portanto, pode-se dizer que o trombo intravascular pulmonar generalizado e a angiogênese induzida por COVID-19 podem levar à hipertensão pulmonar, que pode resultar em um *strain* súbito do ventrículo direito. Dessa forma, podemos estimar que o estresse do ventrículo direito pode aparecer nos achados do ECG e da ecocardiografia. Em consonância com essa sugestão, mostramos que o estresse ventricular direito pode ser determinado pelo ECG em pacientes com COVID-19 pela primeira vez. Nossos achados também foram corroborados por um estudo recente que demonstrou prevalência de dilatação ventricular direita, também detectada por exame ecocardiográfico, entre os pacientes hospitalizados com infecção por COVID-19.^13^ Entretanto, as relações entre dilatação ventricular direita, dano cardíaco, bem como biomarcadores de inflamação e trombose não foram investigados no estudo acima mencionado.

Nosso estudo tem algumas limitações que precisam ser declaradas. Primeiro, este é um estudo de centro único com um tamanho de amostra pequeno. Em segundo lugar, como é um estudo retrospectivo, investigações laboratoriais específicas, como outros biomarcadores trombóticos e cardíacos ou exames ecocardiográficos, e monitoração de ECG para possível reversão dos sinais do ECG, não estavam disponíveis. Terceiro, como o estudo incluiu apenas os pacientes com COVID-19, os pacientes sem COVID-19 não puderam ser comparados, especialmente em relação aos sinais do ECG.

## Conclusões

Que seja de nosso conhecimento, o presente estudo é o primeiro que mostra as manifestações do *strain* do ventrículo direito no ECG e sua relação com a inflamação e trombose em pacientes com pneumonia por COVID-19. Além disso, os achados deste estudo revelam que a infecção por COVID-19 afeta o coração, especialmente através do lado direito do coração. Ao fazer isso, nosso estudo eventualmente sugere que o ECG, que é um teste viável, econômico, seguro e rápido, pode ser utilizado tanto na avaliação quanto no monitoramento dos pacientes com COVID-19 em termos de dano cardíaco, gravidade da inflamação e talvez prognóstico. Finalmente, nossos achados precisam ser validados através de estudos clínicos prospectivos e maiores.
