# Supplementary material for: Relação entre os Sinais de Strain do Ventrículo Direito no Eletrocardiograma e Níveis de Biomarcadores Associados à Gravidade da Pneumonia por COVID-19
Source: Arq Bras Cardiol. 2021 Jun 25;117(4):728–36. [Article in Portuguese] doi: 10.36660/abc.20200724 (PMC8528369; doi:10.36660/abc.20200724)
Supplement: Translation

# Translation: Relationship between Right Ventricular Strain Signs in Electrocardiography and Levels of Biomarkers Associated with COVID-19 Pneumonia Severity

**Authors:** Veli Polat, Evin Bozcali, Kadriye Kart Yasar, Hayat Kumbasar Karaosmanoglu, Ibrahim Faruk Akturk

**Affiliations:** 1 Turkey Department of Cardiology, Bakirkoy Dr. Sadi Konuk Training and Research Hospital, Istanbul - Turkey; 2 Turkey Department of Cardiology, Prof. Dr. Cemil Tascioglu City Hospital, Istanbul - Turkey; 3 Turkey Department of Infectious Diseases and Clinical Microbiology, Bakirkoy Dr. Sadi Konuk Training and Research Hospital, Istanbul - Turkey

**Keywords:** COVID-19, Betacoronavirus, Cardiovascular Diseases, Ventricular Function Right, Stress, Eletrocardiography/methods, Biomarkers, Pneumonia/complications

## Abstract

**Background:**

The novel coronavirus disease (COVID-19) may lead to severe disease that can cause death. COVID-19 is known to affect the cardiovascular system. Early detection of the progression to the severe disease stage that affects the cardiovascular system may play a critical role in the treatment of COVID-19.

**Objectives:**

To explore the possible relationship between the COVID-19 pneumonia and right ventricular strain findings on electrocardiography (ECG).

**Methods:**

We conducted a retrospective study of 141 hospitalized patients with COVID-19. Spearman’s correlation and logistic regression analyses were applied to assess relationships between ECG manifestations of right ventricular strain and levels of biomarkers and other laboratory and chest imaging findings. The significance level was considered as < 0.05.

**Results:**

The ECG signs of right ventricular stress were significantly more frequent and the levels of fibrinogen, CRP, and ferritin were significantly higher in COVID-19 patients with elevated levels of hs-cTnI, procalcitonin and D-dimer. The univariate analysis showed there are significant relations between the presence of bilateral pneumonia, most of the ECG signs of right ventricular strain and cardiac injury and inflammatory and thrombotic biomarkers. The multivariate analysis revealed that ST-segment elevation in V_1_and the S_1_Q_3_T_3_pattern are independent predictors of cardiac damage (odds ratio=0.23; 95% CI, 0.06 to 0.90; p=0.035) and elevated procalcitonin levels (odds ratio=0.19; 95% CI, 0.06 to 0.62; p=0.006), respectively.

**Conclusion:**

The findings of the present study suggest that right heart damage is prevalent in COVID-19. In addition, our study shows the clinical value of ECG in evaluating and monitoring the patients with COVID-19 pneumonia.

## Introduction

The 2019 novel coronavirus disease (COVID-19) pneumonia, which first emerged in Wuhan, China’s Hubei province, in December 2019, has rapidly spread across the world. Although respiratory symptoms are primary clinical signs of COVID-19, several patients also develop cardiovascular injury, which is predominantly detected as an increase in high-sensitivity cardiac troponin assays.^1^ Moreover, it has been demonstrated that the COVID-19 pneumonia has a worse clinical course in patients with cardiovascular disease.^2^ In this context, disclosing the ways in which the novel coronavirus affects the cardiovascular system is crucial to the treatment of these patients in a timely and efficient fashion and for the reduction of ominous outcomes such as death.

In the present study, we assess the abnormal electrocardiogram (ECG) manifestations in COVID-19. Specifically, we aimed to investigate the possible relationship between the severity of COVID-19 pneumonia and the right ventricular strain findings in the ECG. Additionally, we intent to explore possible relationships between the electrocardiographic abnormalities, cardiac injury, as well as elevated biomarkers of inflammation and thrombosis in COVID-19 patients.

## Materials and methods

### Study Patients

This retrospective, single-center and observational study was conducted at Bakirkoy Dr. Sadi Konuk Training and Research Hospital. Between April 1 and April 30, 2020, 141 consecutive hospitalized patients who were diagnosed with COVID-19 pneumonia according to the World Health Organization interim guidance^3^ were enrolled in the present study. The diagnosis of COVID-19 infection was also confirmed by the positive result of real-time reverse-transcriptase-polymerase chain reaction analysis of the patient’s nasal or pharyngeal swab samples. The ECGs of the patients were obtained on admission. All of the study patients were adults (over 18 years old) and the diagnosis of pulmonary embolism was excluded by the findings of contrast-enhanced chest computed tomography (CT) in all patients.

### Data Collection

The demographic, epidemiological, clinical, and radiological data, as well as comorbidities and laboratory findings at admission were recorded in the electronic medical records for each study patient. Laboratory data included the high-sensitive cardiac troponin I (hs-cTnI), creatine kinase-myocardial brain fraction (CK-MB), complete blood count, C-reactive protein (CRP), procalcitonin, fibrinogen, D-dimer, ferritin, kidney and liver function tests. All of the patients were clinically followed up to May 25, 2020. Two investigators controlled all study data.

Elevated hs-cTnI levels, which is the sign of cardiac damage, was considered when >the 99^th^percentile upper reference limit. The levels of procalcitonin and D-dimer were considered elevated when ≥ 0.5 ng/mL and ≥ 0.5 µg/mL, respectively.^4^

### Electrocardiographic Evaluation

The 12-lead ECGs obtained at the time of admission were independently interpreted regarding the right ventricular strain signs by two experienced cardiologists who were blinded to patient data. ECG signs were determined based on the ECG manifestations due to right ventricular stress secondary to pulmonary hypertension in pulmonary embolism.^5 , 6^ The following ECG signs of right ventricular strain were evaluated: (1) sinus tachycardia (heart rate > 100 beats/minute),^5^ (2) incomplete or complete right bundle branch block (RBBB),^5^ (3) T-wave inversion in leads V_1_to V_4,_^5^ (4) S_1_Q_3_T_3_pattern,^5^ (5) Qr in lead V_1_(existence of a prominent Q wave ≥ 0.2 millivolts while QRS duration is <120 milliseconds)^6^ , (6) ST-segment elevation ≥0.1 millivolt in lead V_1._^6^

### Statistical Analysis

Descriptive statistics were performed using mean ± standard deviation (SD) for normally-distributed or median and interquartile ranges (IQR) for skewed-distributed continuous variables and percentage for categorical variables. The distribution of variables was assessed by the Kolmogorov-Smirnov test. Continuous variables were compared by the independent-samples *t* test when normally distributed or the Mann-Whitney-U test when not normally distributed. Categorical variables were compared with the chi-square (X^2^ ) test or Fisher’s exact test, where appropriate. Correlations between the levels of hs-cTnI, procalcitonin, D-dimer, CRP, fibrinogen, ferritin and neutrophil count were assessed by Spearman’s correlation coefficient analysis. The univariate logistic regression analysis was performed to test the relationship between the variables and the elevated hs-cTnI, procalcitonin and D-dimer levels. Additionally, odds ratios and 95% confidence intervals (CIs) were calculated for each variable. The multivariate logistic regression analysis was performed to assess the independent predictors of cardiac damage (elevated levels of hs-cTnI), elevated levels of procalcitonin and D-dimer. The variables that were significant in the univariate analysis were included in the multivariate analysis. A p value < 0.05 was considered as statistically significant. All statistical analyses were carried out using IBM SPSS software version 26.0.

## Results

One-hundred and forty-one consecutive patients hospitalized with COVID-19 pneumonia were analyzed in the present study. The study patients’ median age was 64 years (range, 26-91 years), and 84 (59.6%) were males. Five patients died during hospital stay and 136 patients were discharged. Comorbidities, electrocardiographic signs of right ventricular strain, radiographic findings, and the clinical outcome of all study patients are shown in Table 1 .

**Table 1:** – Comorbidities, clinical outcome, electrocardiographic and radiographic findings of patients with COVID-19 pneumonia

	Patients (n=141)
Smoking	21 (14.9%)
Comorbidities	
Hypertension	44 (31.2%)
Diabetes	36 (25.5%)
Coronary artery disease	27 (19.1%)
Chronic obstructive pulmonary disease	5 (3.5%)
Malignancy	2 (1.4%)
ECG signs	
Sinus tachycardia	87 (61.7%)
Incomplete RBBB	62 (44.0%)
Complete RBBB	28 (19.9%)
T-wave inversion in V1 to V4	37 (26.2%)
S_1_Q_3_T_3_pattern	52 (36.9%)
Qr in V_1_	35 (24.8%)
ST-segment elevation in V_1_	54 (38.3%)
Chest computed tomography findings	7 (5%)
Unilateral pneumonia	55 (39.0%)
Bilateral pneumonia	86 (61.0%)
Clinical outcome	35 (24.8%)
In-hospital death	5 (3.5%)

*ECG: electrocardiogram; RBBB: right bundle branch block.*

### Clinical and Electrocardiographic Characteristics

Table 2 shows a summary of the comparison of the demographic data, signs of ECG, CT findings, clinical outcome, and laboratory data by categorizing as elevated or normal the levels of hs-cTnI, procalcitonin and D-dimer in patients with COVID-19 pneumonia.. Smoking status, gender and the presence of comorbidities were similar between patients with elevated or normal levels of hs-cTnI, procalcitonin and D-dimer.

**Table 2:** – Comparison of the characteristics of patients with COVID-19 pneumonia according to elevated or normal hs-cTnI, procalcitonin and D-dimer levels

Characteristics	Normal hs-cTnI (n = 69)	Elevated hs-cTnI (n = 72)	p Value	Procalcitonin < 0.5 ng/mL (n = 74)	Procalcitonin ≥ 0.5 ng/mL (n = 67)	p Value	D-dimer < 0.5 µg/mL (n = 39)	D-dimer ≥ 0.5 µg/mL (n = 102)	p Value
Age	62.6 ± 12.3	63.4 ± 12.5	0.69^†^	60.1 ± 12.1	66.3 ± 11.1	0.003^†^	57.0 ± 12.6	65.3 ± 11.5	< 0.001^†^
Female	29 (42%)	28 (38.9%)	0.70^∗^	29 (39.2%)	28 (41.8%)	0.75^∗^	13 (33.3%)	44 (43.1%)	0.289^∗^
ECG signs									
Sinus tachycardia	34 (49.3%)	53 (73.6%)	0.003^∗^	30 (40.5%)	57 (85.1%)	< 0.001^∗^	9 (23.1%)	78 (76.5%)	< 0.001^∗^
Incomplete RBBB	21 (30.4%)	41 (56.9%)	0.002^∗^	28 (37.8%)	34 (50.7%)	0.123^∗^	16 (41%)	46 (45.1%)	0.66^∗^
Complete RBBB	8 (11.6%)	20 (27.8%)	0.016^∗^	4 (5.4%)	24 (35.8%)	< 0.001^∗^	2 (5.1%)	26 (25.5%)	0.007^∗^
T inversion in V1 to V4	3(4.3%)	34 (47.2%)	< 0.001^∗^	4 (5.4%)	33(49.3%)	< 0.001^∗^	1 (2.6%)	36 (35.3%)	< 0.001^∗^
S_1_Q_3_T_3_pattern	14 (20.3%)	38 (52.8%)	< 0.001^∗^	8 (10.8%)	44 (65.7%)	< 0.001^∗^	2 (5.1%)	50 (49%)	< 0.001^∗^
Qr in V_1_	1 (1.4%)	34 (47.2%)	< 0.001^∗^	3 (4.1%)	32 (47.8%)	< 0.001^∗^	1 (2.6%)	34 (33.3%)	< 0.001^∗^
ST elevation in V_1_	5 (7.2%)	49 (68.1%)	< 0.001^∗^	5 (6.8%)	49 (73.1%)	< 0.001^∗^	0 (0.0%)	54 (52.9%)	< 0.001^∗^
Chest CT findings									
Unilateral pneumonia	40 (58%)	15 (20.8%)	< 0.001^∗^	52 (70.3%)	3 (4.5%)	< 0.001^∗^	31 (79.5%)	24 (23.5%)	< 0.001^∗^
Bilateral pneumonia	29 (42%)	57 (79.2%)		22 (29.7%)	64 (95.5%)		8 (20.5%)	78 (76.5%)	
Clinical outcome									
In-hospital death	0 (0.0%)	5 (6.9%)	0.026^γ^	0 (0.0%)	5 (7.5%)	0.017^γ^	0 (0.0%)	5 (4.9%)	0.322^γ^
Laboratory data									
Leukocyte count ×10^3^/μL	6.6 (5.6-7.8)	6.9 (5.3-8.9)	0.66^‡^	6.5 (5.3-8.2)	7.3 (5.7-8.6)	0.104^‡^	6.8 (5.4-7.7)	6.7 (5.6-6.6)	0.269^‡^
Neutrophil count ×10^3^/μL	4.9 (3.3-5.9)	4.7 (3.9 – 6.8)	0.22^‡^	4.7 (3.2-5.7)	5.4 (4-7.8)	0.004^‡^	4.9 (3.2-5.8)	4.9 (3.8-6.8)	0.203^‡^
Neutrophil/ Leukocyte ratio, %	70.5 ± 8.9	74.0 ± 10.2	0.033^†^	69.6 ± 9.2	75.3 ± 9.5	< 0.001^†^	69.4 ± 9.4	73.4± 9.7	0.03^†^
Lymphocyte count ×10^3^/μL	1.1 (0.9- 1.3)	0.9 (0.8-1.2)	0.006^‡^	1.05 (0.9-1.1)	0.96 (0.9-1.2)	0.019^‡^	1.10 (0.9-1.6)	0.97 (1-1.2)	0.009^‡^
Lymphocyte / Leukocyte ratio, %	15.6 (13.6 -20)	15 (10-19.7)	0.06^‡^	16.7 (14.3-20)	13.6 (9.5 -19)	0.003^‡^	17 (14-17)	15 (10-18)	0.007^‡^
Platelets ×10^3^/μL	220 (168 – 256)	221 (163-285)	0.98^‡^	219 (167-256)	220 (166-306)	0.82^‡^	218 (163-243)	220 (167-291)	0.411^‡^
Creatinine (mg/dL)	0.86 (0.7-1)	0.98 (0.8 -1.2)	0.16^‡^	0.86 (0.7-1.1)	0.98 (0.8-1.3)	0.09^‡^	0.83 (0.7-1)	0.97 (0.8-1.2)	0.13^‡^
ALT (U/L)	31 (25-45)	31.5 (20-49)	0.74^‡^	30 (21-45)	32 (23-47)	0.55^‡^	31 (25-43)	31 (21-45)	0.32^‡^
AST (U/L)	30 (24-38)	35.5 (26-47)	0.11^‡^	30 (23-39)	36 (26-46)	0.23^‡^	30 (23-40)	34 (26-44)	0.37^‡^
Fibrinogen, mg/dL	472 (413-542)	590 (505-736)	< 0.001^‡^	449 (409-507)	626 (550-740)	< 0.001^‡^	415 (396-428)	577 (505-683)	< 0.001^‡^
D-dimer, µg/mL	0.45 (0.2-0.9)	1.40 (0.7-3.7)	< 0.001^‡^	0.41 (0.2-0.8)	2.04 (1-3.8)	< 0.001^‡^	0.24 (0.2-0.3)	1.20 (0.7-2.8)	< 0.001^‡^
CRP, mg/L	58 (44-88)	136 (89-203)	< 0.001^‡^	54 (43-87)	153 (153-204)	< 0.001^‡^	49 (41-61)	114 (77-179)	< 0.001^‡^
Ferritin, µg/L	294 (236-400)	612 (397-975)	< 0.001^‡^	282 (232- 382)	662 (450-980)	< 0.001^‡^	254 (226-292)	532 (356-806)	< 0.001^‡^
Procalcitonin, ng/mL	0.06 (0.02-0.38)	2.76 (0.31-6.69)	< 0.001^‡^	0.055 (0.02-0.07)	2.76 (1-7.7)	< 0.001^‡^	0.05 (0.02-0.06)	0.96 (0.07-5.30)	< 0.001^‡^
CK-MB, ng/mL	3 (2-3)	8.5 (5-15.5)	< 0.001^‡^	3 (2-4)	9 (4-16)	< 0.001^‡^	3 (2-4)	5.5 (3-12)	< 0.001^‡^
hs-cTnI, ng/L	15 (12-16)	62.5 (26-348)	< 0.001^‡^	16 (13-18)	67 (18-356)	< 0.001^‡^	16 (13-16)	31 (16-173)	< 0.001^‡^

*Data are presented as mean ± SD or median (IQR) or N. (%). ECG: electrocardiogram; hs-cTnI: highly-sensitive cardiac troponin I; CT: computed tomography; ALT: Alanine aminotransferase; AST: Aspartate aminotransferase; CRP: C-reactive protein; CK-MB: creatine kinase-myocardial brain fraction. † Compared using the independent sample t test, ‡Compared using the Mann-Whitney U test, ∗Compared using the X2 test, γ Compared using Fisher’s exact test.*

In patients with elevated levels of hs-cTnI, procalcitonin, and D-dimer, the ECG signs of right ventricular stress, including sinus tachycardia, complete RBBB, T-wave inversion in V1-V4, S1Q3T3 pattern, Qr morphology in lead V1 and ST-segment elevation in V1 were significantly more prevalent when compared to the patients with normal levels of hs-cTnI, procalcitonin, and D-dimer. The ratio of bilateral pneumonia based on the findings of the chest CT was significantly higher in patients with elevated levels of hs-cTnI, procalcitonin, and D-dimer. Patients with elevated levels of hs-cTnI, procalcitonin, and D-dimer also had significantly higher levels of fibrinogen, CRP, ferritin, CK-MB and a higher neutrophil-to-leukocyte ratio, while they had significantly lower lymphocyte count ( Table 2 ).

In-hospital mortality was significantly higher in the patients with elevated hs-cTnI and procalcitonin levels. Incomplete RBBB was significantly frequent in the patients who only had cardiac damage. Patients with elevated procalcitonin levels had significantly higher neutrophil count than those with normal procalcitonin levels. Patients with elevated levels of procalcitonin and D-dimer were significantly older, and their lymphocyte-to-leukocyte ratios were significantly lower ( Table 2 ).

Moreover, patients with cardiac damage presented with significantly higher levels of D-dimer and procalcitonin than those without cardiac damage. D-dimer and hs-cTnI levels were significantly increased in patients with elevated procalcitonin levels, when compared to patients with normal procalcitonin levels. Procalcitonin and hs-cTnI levels were also found to be significantly higher in patients with elevated D-dimer levels, when compared to those with normal D-dimer levels ( Table 2 ).

### Univariate and Multivariate Logistic Regression Analyses

The univariate logistic regression analysis showed that sinus tachycardia, incomplete RBBB, complete RBBB, T-wave inversion in V_1_-V_4_, S_1_Q_3_T_3_pattern, Qr morphology in V_1_, ST-segment elevation in V_1_, presence of bilateral pneumonia, neutrophil count, CRP, procalcitonin, ferritin, fibrinogen, D-dimer and CK-MB levels were significantly associated with cardiac damage in patients with COVID-19 pneumonia ( Table 3 ). However, at the multivariate logistic regression analysis, only the ST-segment elevation in V_1_was determined as an independent predictor of cardiac damage in patients with COVID-19 pneumonia (odds ratio=0.23; 95% CI, 0.06 to 0.90; p=0.035).

**Table 3:** – Determinants of cardiac damage in patients with COVID-19 by univariate and multivariate logistic regression analyses

	Univariate		Multivariate		

	OR (95% CI)		p Value	OR (95% CI)	p Value
Sinus tachycardia	0.35 (0.17-0.70)	0.003			
Incomplete RBBB	0.33 (0.17-0.66)	0.002			
Complete RBBB	0.34 (0.14-0.84)	0.019			
T inversion in V_1_-V_4_	0.05 (0.01-0.18)	< 0.001			
S_1_Q_3_T_3_pattern	0.23 (0.11-0.48)	< 0.001			
Qr in V_1_	0.02 (0.00-0.12)	< 0.001			
ST elevation in V_1_	0.04 (0.01-0.10)	< 0.001		0.23 (0.06-0.90)	0.035
Presence of bilateral pneumonia	0.19 (0.09-0.40)	< 0.001			
Neutrophil count	1.04 (1.00-1.08)	0.036			
Fibrinogen, mg/dL	1.01 (1.01-1.01)	< 0.001			
D-dimer, µg/mL	3.14 (1.91-5.17)	< 0.001			
CRP, mg/L	1.02 (1.01-1.03)	< 0.001			
Ferritin, µg/L	1.01 (1.00-1.01)	< 0.001			
Procalcitonin, ng/mL	2.03 (1.45-2.83)	< 0.001			
CK-MB, ng/mL	2.09 (1.58-2.76)	< 0.001			

*OR: Odds ratio; CI: confidence interval; RBBB: right bundle branch block; CRP: C-reactive protein.*

The patients’ age, complete RBBB, T-wave inversion in V_1_-V_4_, S_1_Q_3_T_3_pattern, Qr morphology in V_1_, ST-segment elevation in V_1_, presence of bilateral pneumonia, lymphocyte-to-leukocyte ratio, neutrophil count, neutrophil-to-leukocyte ratio, CRP, ferritin, fibrinogen, D-dimer, hs-cTnI, and CK-MB levels were significantly associated with the elevated procalcitonin levels in the univariate logistic regression analysis. The multivariate logistic regression analysis revealed that the S_1_Q_3_T_3_pattern (odds ratio=0.19; 95% CI, 0.06 to 0.62; p=0.006), neutrophil count (odds ratio=1.35; 95% CI, 1.07 to 1.71; p=0.011) and presence of bilateral pneumonia (odds ratio=0.15; 95% CI, 0.03 to 0.84; p=0.031) were independently associated with the elevated levels of procalcitonin ( Table 4 ).

**Table 4:** – Determinants of the elevated levels of procalcitonin (≥ 0.5 ng/mL) in patients with COVID-19 by univariate and multivariate logistic regression analyses

	Univariate		Multivariate		

	OR (95% CI)		p Value	OR (95% CI)	p Value
Age	1.04 (1.01-1.08)	0.004			
Complete RBBB	0.10 (0.03-0.32)	< 0.001			
T inversion in V_1_-V_4_	0.06 (0.02-0.18)	< 0.001			
S_1_Q_3_T_3_pattern	0.06 (0.03-0.15)	< 0.001		0.19 (0.06-0.62)	0.006
Qr in V_1_	0.05 (0.01-0.16)	< 0.001			
ST elevation in V_1_	0.03 (0.01-0.08)	< 0.001			
Presence of bilateral pneumonia	0.02 (0.01-0.07)	< 0.001		0.15 (0.03-0.84)	0.031
Neutrophil count	1.27 (1.09-1.48)	0.002		1.35 (1.07-1.71)	0.011
Neutrophil/Leukocyte ratio	1.07 (1.03-1.11)	0.001			
Lymphocyte/Leukocyte ratio	0.93 (0.88-0.98)	0.009			
Fibrinogen, mg/dL	1.02 (1.01-1.02)	< 0.001			
D-dimer, µg/mL	8.64 (3.67-20.35)	< 0.001			
CRP, mg/L	1.03 (1.02-1.04)	< 0.001			
Ferritin, µg/L	1.01 (1.01-1.01)	< 0.001			
CK-MB, ng/mL	1.35 (1.18-1.53)	< 0.001			
hs-cTnI, ng/L	1.02 (1.01-1.02)	< 0.001			

*OR: Odds ratio; CI: confidence interval; RBBB: right bundle branch block; CRP: C-reactive protein.*

According to the univariate analysis, the patients’ age, presence of bilateral pneumonia, the lymphocyte-to-leukocyte ratio, neutrophil and lymphocyte counts, levels of CRP, ferritin, fibrinogen, procalcitonin, hs-cTnI, CK-MB and the presence of ECG signs, including complete RBBB, T-wave inversion in V_1_-V_4_, S_1_Q_3_T_3_pattern, and Qr morphology in V_1_were significantly associated with the elevated levels of D-dimer ( Table 5 ). Nonetheless, among these determinants, only fibrinogen was found to be an independent predictor of the elevated levels of D-dimer in the multivariate analysis (odds ratio=1.05; 95% CI, 1.03 to 1.07; p<0.001).

**Table 5:** – Determinants of the elevated levels of D-dimer (≥ 0.5 µg/mL) in patients with COVID-19 by univariate and multivariate logistic regression analyses

	Univariate		Multivariate		

	OR (95% CI)		p Value	OR (95% CI)	p Value
Age	1.06 (1.02-1.10)	0.001			
Complete RBBB	0.16 (0.04-0.70)	0.015			
T inversion in V_1_-V_4_	0.05 (0.01-0.37)	0.003			
S_1_Q_3_T_3_pattern	0.06 (0.01-0.25)	< 0.001			
Qr in V_1_	0.05 (0.01-0.40)	0.004			
Presence of bilateral pneumonia	0.08 (0.03-0.20)	< 0.001			
Neutrophil count	1.04 (1.00-1.09)	0.033			
Lymphocyte count	0.41 (0.17-0.96)	0.041			
Lymphocyte/Leukocyte ratio	0.93 (0.88-0.98)	0.007			
Fibrinogen, mg/dL	1.04 (1.02-1.06)	< 0.001		1.05 (1.03-1.07)	< 0.001
Procalcitonin, ng/mL	17.73 (2.82-111.30)	0.002			
CRP, mg/L	1.05 (1.03-1.07)	< 0.001			
Ferritin, µg/L	1.01 (1.01-1.02)	< 0.001			
CK-MB, ng/mL	1.34 (1.12-1.61)	0.001			
hs-cTnI, ng/L	1.10 (1.03-1.18)	0.006			

*OR: Odds ratio; CI: confidence interval; RBBB: right bundle branch block; CRP: C-reactive protein.*

The Spearman’s correlation analysis also revealed that neutrophil count, hs-cTnI, procalcitonin, D-dimer, CRP, fibrinogen and ferritin levels were significantly and positively correlated with each other ( Table 6 ).

**Table 6:** – Spearman’s correlations of biomarkers with each other in patients with COVID-19

	hs-cTnI		Procalcitonin		D-dimer		CRP		Neutrophil count		Fibrinogen		Ferritin	

	r	p	r	p	r	p	r	p	r	p	r	p	r	p
hs-cTnI	1	.	0.666	< 0.001	0.633	< 0.001	0.599	< 0.001	0.175	0.04	0.618	< 0.001	0.625	< 0.001
Procalcitonin	0.666	< 0.001	1	.	0.776	< 0.001	0.811	< 0.001	0.213	0.01	0.748	< 0.001	0.841	< 0.001
D-dimer	0.633	< 0.001	0.776	< 0.001	1	.	0.821	< 0.001	0.172	0.04	0.924	< 0.001	0.842	< 0.001
CRP	0.599	< 0.001	0.811	< 0.001	0.821	< 0.001	1	.	0.271	0.001	0.811	< 0.001	0.962	< 0.001
Neutrophil count	0.175	0.04	0.213	0.01	0.172	0.04	0.271	0.001	1	.	0.174	0.04	0.222	0.008
Fibrinogen	0.618	< 0.001	0.748	< 0.001	0.924	< 0.001	0.811	< 0.001	0.174	0.04	1	.	0.832	< 0.001
Ferritin	0.625	< 0.001	0.841	< 0.001	0.842	< 0.001	0.962	< 0.001	0.222	0.008	0.832	< 0.001	1	.

*CRP; C-reactive protein; hs-cTnI: highly-sensitive cardiac troponin I; r: Spearman’s correlation coefficient.*

## Discussion

Cardiac damage detected by the increase of cardiac biomarkers in COVID-19 has been associated with a fatal prognosis. However, the pathophysiological mechanisms underlying cardiac damage associated with COVID-19 have not been clearly demonstrated. The possible mechanisms that other studies have suggested to explain the myocardial damage caused by COVID-19 are myocarditis due to direct infection with SARS-CoV-2 or secondary to the vigorous systemic inflammatory response induced by COVID-19, plaque instability that leads to acute coronary syndrome, and hypoxemia due to respiratory failure.^7 , 8^ Recently, pulmonary microvascular thrombosis has been reported with diffuse alveolar damage in COVID-19 pneumonia.^9 , 10^

McGonagle et al. have proposed a pulmonary intravascular coagulopathy model to explain myocardial injury and cardiovascular mortality in COVID-19 pneumonia. According to this model, COVID-19 induces diffuse pulmonary intravascular coagulopathy through diffuse lung inflammation, similar to the macrophage activation syndrome. Both the immunothrombosis of the extensive pulmonary vascular bed and the alveolar hypoxemia may trigger right ventricular stress by causing pulmonary hypertension and may play a role in the fatal outcome. In COVID-19 pneumonia, elevated D-dimer levels may reflect wide pulmonary microthrombosis, while elevated cardiac biomarker levels may indicate right ventricular strain triggered by pulmonary hypertension.^11^ The findings of our current study support this model. All of the ECG signs of right ventricular strain in the study, except incomplete RBBB, were significantly more prevalent in COVID-19 patients with cardiac damage and elevated procalcitonin and D-dimer levels. As we expected, all of the ECG signs of right ventricular strain, including incomplete RBBB, were significantly more common in COVID-19 patients with cardiac damage. We also observed a significant and direct association among the levels of hs-cTnI, procalcitonin and D-dimer, neutrophil count, levels of CRP, ferritin and fibrinogen in patients with COVID-19 pneumonia. These results indicate that inflammation, thrombosis and cardiac damage are directly related with each other in COVID-19 patients.

The univariate regression analysis revealed that four of the seven ECG signs of right ventricular strain were significantly associated with cardiac injury and elevated levels of procalcitonin and D-dimer in patients with COVID-19 pneumonia. However, all of these seven ECG signs were significantly associated only with cardiac injury in COVID-19 patients. These findings suggest that the possible cause of elevated hs-cTnI levels or cardiac injury in COVID-19 is right ventricular stress, which is induced by sudden-onset pulmonary hypertension due to diffuse pulmonary vascular thrombosis triggered by COVID-19 pneumonia.

The multivariate analysis revealed that among the ECG signs and other variables, only the ST-segment elevation in V_1_was an independent predictor of cardiac damage in patients with COVID-19 pneumonia. Therefore, the existence of ST-segment elevation in V_1_on the admission ECG may indicate a poor prognosis in patients with COVID-19, when we consider that the cardiac injury is associated with poor prognosis in these patients. Moreover, the existence of ST-segment elevation in V_1_was also significantly associated with elevated procalcitonin levels, according to our results of the regression analyses. The S_1_Q_3_T_3_pattern, which is a well-known electrocardiographic manifestation of acute right heart strain, particularly developed due to massive pulmonary embolism, was found to be one of the independent predictors of elevated procalcitonin levels in patients with COVID-19 pneumonia in our multivariate regression analyses. Unsurprisingly, the other two independent predictors of elevated procalcitonin levels in COVID-19 patients were the presence of bilateral pneumonia, and neutrophil count. As both of these predictors are related to the extensiveness and severity of the disease, we suggest that the S_1_Q_3_T_3_pattern may also reflect disease extent and severity.

Interestingly, there was no relationship between the patients’ age and cardiac damage. Yet, the univariate regression analysis demonstrated a positive relationship between age and elevated levels of procalcitonin and D-dimer. The multivariate analysis indicated that fibrinogen was the only independent predictor of elevated D-dimer levels. However, none of the ECG signs of the right ventricular strain was an independent predictor of elevated D-dimer levels. Although elevated D-dimer levels may be the reflection of the diffuse pulmonary intravascular thrombosis in COVID-19, elevated D-dimer levels are not specific and can occur due to many other conditions. Therefore, we concluded that we did not find an independent relationship between the elevated D-dimer levels and the ECG signs on the multivariate analysis.

Very recently, Ackermann e al.^12^ have published an article that investigated the fundamental pathological mechanisms of severe COVID-19 infection by examining the morphological and molecular changes of the lungs obtained during the autopsy of patients who died due to COVID-19 infection. They have reported that both COVID-19 and influenza exhibits diffuse alveolar damage and infiltrating perivascular lymphocytes, although COVID-19 is distinct with its three angiocentric characteristics. These characteristics are the extensive vascular thrombosis with microangiopathy and occlusion of alveolar capillaries, vascular angiogenesis via intussusceptive angiogenesis, and endothelial cell damage possibly due to the direct invasion by SARS-CoV-2.^12^ Their findings support ours by showing widespread pulmonary vascular thrombosis, which is accompanied by endothelial injury and angiogenesis caused by COVID-19. Therefore, it can be stated that widespread pulmonary intravascular thrombus and angiogenesis induced by COVID-19 may lead to pulmonary hypertension, which may result in sudden right ventricular strain. Therefore, it can be estimated that right ventricular stress may appear in the ECG and echocardiography findings. In agreement with this suggestion, we have shown that right ventricular stress can be determined by the ECG in patients with COVID-19 for the first time. Our findings were also supported by a recent study that demonstrated a prevalence of right ventricular dilation, which was also detected by echocardiographic examination, in hospitalized patients with COVID-19 infection.^13^ However, the relationships between right ventricular dilation, cardiac damage and biomarkers of both inflammation and thrombosis were not investigated in the aforementioned study.

Our study has some limitations that need to be stated. First, this is a single-center study, with a small sample size. Second, as a retrospective study, specific laboratory investigations such as other thrombotic and cardiac biomarkers or echocardiographic examinations, and ECG monitoring for possible reversion of the ECG signs were not available. Third, as the study included patients with COVID-19 only, patients without COVID-19 could not be compared, especially regarding ECG signs.

## Conclusions

To the best of our knowledge, the present study is the first one to show the right ventricular strain manifestations on the ECG and its relationship with inflammation and thrombosis in patients with COVID-19 pneumonia. Additionally, the findings of this study reveal that COVID-19 infection affects the heart, especially through the right side of the heart. In doing so, our study eventually suggests that the ECG, which is a feasible, cost-effective, safe and rapid test, can be used in both the assessment and monitoring of patients with COVID-19 in terms of cardiac damage, severity of the inflammation and perhaps prognosis. Finally, our findings need to be validated by prospective and larger clinical trials.
